# Supplementary material for: Comparison of spatiotemporal characteristics of the COVID-19 and SARS outbreaks in mainland China
Source: BMC Infect Dis. 2020 Oct 30;20:805. doi: 10.1186/s12879-020-05537-y (PMC7598229; doi:10.1186/s12879-020-05537-y)

Additional file 2: Spatiotemporal clustering of COVID-19 incident cases in stage1 from January 20 to February 6, 2020 **(a)** and stage2 from February 7 to March 4, 2020 **(b)** (including Hubei province). We drew this figure using ArcGIS software v10.2.2.

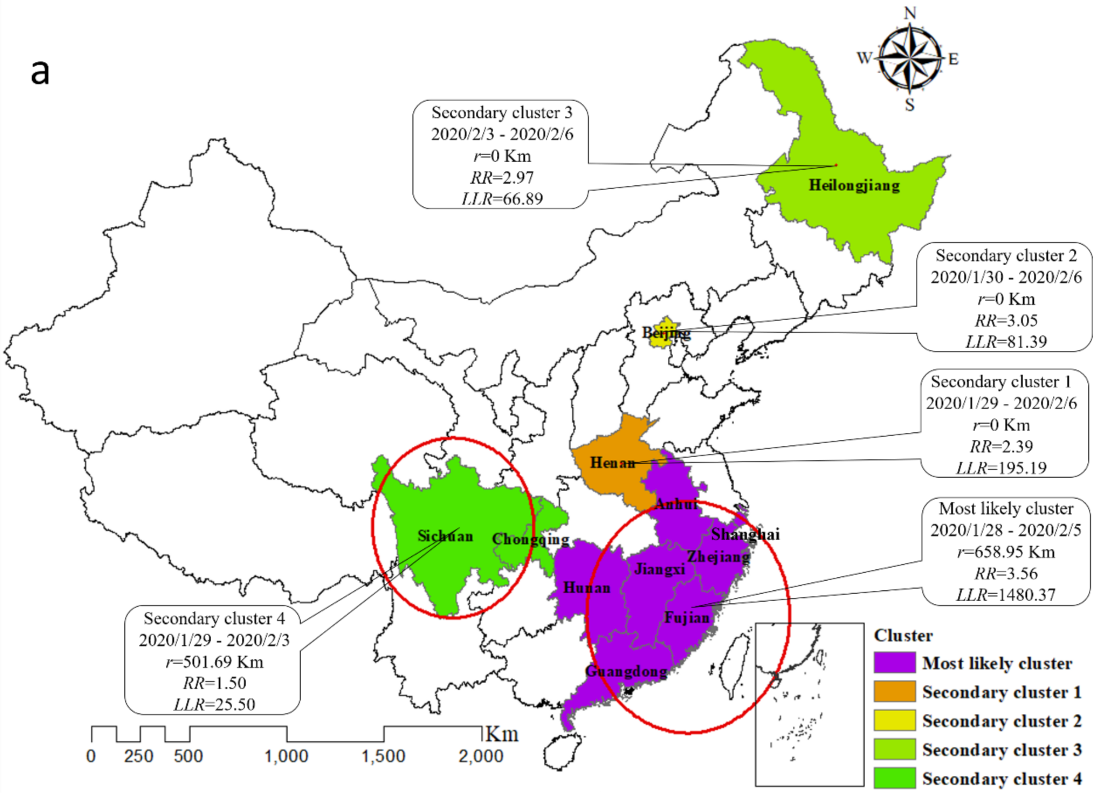

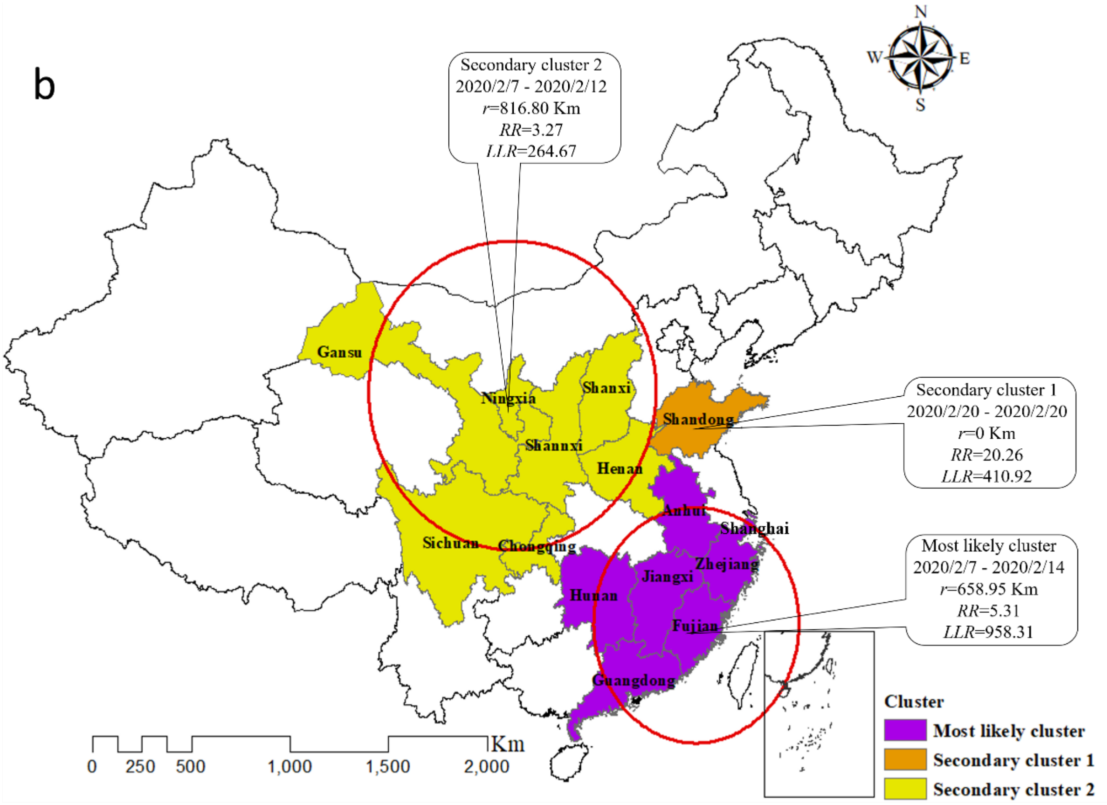

Supplement: Supplementary file 2 — Additional file 2 Spatiotemporal clustering of COVID-19 incident cases in stage1 from January 20 to February 6, 2020 (a) and stage2 from February 7 to March 4, 2020 (b) (excluding Hubei province). We drew this figure using ArcGIS software v10.2.2. [file 12879_2020_5537_MOESM2_ESM.pdf]
